# Downregulation of ubiquitin inhibits the proliferation and radioresistance of non-small cell lung cancer cells *in vitro* and *in vivo*

**DOI:** 10.1038/srep09476

**Published:** 2015-03-30

**Authors:** Yiting Tang, Yangyang Geng, Judong Luo, Wenhao Shen, Wei Zhu, Cuicui Meng, Ming Li, Xifa Zhou, Shuyu Zhang, Jianping Cao

**Affiliations:** 1 School of Radiation Medicine and Protection and Jiangsu Provincial Key Laboratory of Radiation Medicine and Protection, Medical College of Soochow University, Suzhou 215123, China; 2 Collaborative Innovation Center of Radiation Medicine of Jiangsu Higher Education Institutions and School for Radiological and Interdisciplinary Sciences (RAD-X), Soochow University, Suzhou 215123, China; 3 Department of Radiotherapy, Changzhou Tumor Hospital, Soochow University, Changzhou. 213001, China

## Abstract

Radioresistance has been an important factor in restricting efficacy of radiotherapy for non-small cell lung cancer (NSCLC) patients and new approaches to inhibit cancer growth and sensitize irradiation were warranted. Despite the important role of ubiquitin/proteasome system (UPS) during cancer progression and treatment, the expression and biological role of ubiquitin (Ub) in human NSCLC has not been characterized. In this study, we found that ubiquitin was significantly overexpressed in 75 NSCLC tissues, compared to their respective benign tissues by immunohistochemistry (*P* < 0.0001). Knock-down of ubiquitin by mixed shRNAs targeting its coding genes *ubiquitin B* (*UBB*) and *ubiquitin C* (*UBC*) suppressed the growth and increased the radiosensitivity in NSCLC H1299 cells. Apoptosis and γ H2AX foci induced by X-ray irradiation were enhanced by knock-down of ubiquitin. Western blot and immunostaining showed that knock-down of ubiquitin decreased the expression and translocation of NF-κB to the nucleus by reduced phospho-IκBα after irradiation. Suppression of ubiquitin decreased the proliferation and radioresistance of H1299 transplanted xenografts in nude mice by promoting apoptosis. Taken together, our results demonstrate the critical role of ubiquitin in NSCLC proliferation and radiosensitivity. Targeting ubiquitin may serve as a potentially important and novel approach for NSCLC prevention and therapy.

Lung cancer is one of the most common malignancies worldwide^1^ and has become the leading cause of cancer related death in some metropolitan area of China. Lung cancer is separated into two morphologic types: non-small cell lung cancer (NSCLC) and small cell lung cancer (SCLC). Approximately 80–85% cases of lung cancer were diagnosed as NSCLC^1^. A number of oncogenes and tumor-suppressor genes are dysregulated in NSCLC and a larger collection of genes mutated or/and dysregulated in NSCLC have also been defined. One of the most characterized mutant genes, epidermal growth factor receptor (EGFR), has been observed with increased expression in 40–80% of NSCLC cells^2^. EGFR inhibition once becomes one of the approaches against cancer cell proliferation^3^. However, most patients with NSCLC are proved to respond poorly to the tyrosine kinase inhibitor (TKI) gefitinib, due to activated mutations in EGFR^4^,^5^. Therefore, exploring new and effective targets to abate the proliferation and facilitate the treatment of lung cancer cell is urgently warranted.

The ubiquitin/proteasome system (UPS), the major cellular protein degradation machinery, has become increasingly recognized as a controller of numerous physiological processes, including signal transduction, DNA repair, chromosome maintenance, transcriptional activation, cell cycle progression, cell survival, and certain immune cell functions^6^. Additionally, anticancer drugs targeting this system such as bortezomib and MG132, in combination with DNA damaging agents have shown promise against various cancers *in vitro* and *in vivo*^7^,^8^. Ubiquitin molecule (Ub) is encoded by four different genes that are highly homologous in eukaryotes. *UBA52* and *RPS27A* genes code for a single copy of ubiquitin fused to the ribosomal proteins L40 and S27a, respectively. The comprehensive protein degradation signal monoubiquitin and polyubiquitin are encoded by *ubiquitin B* (*UBB*) and *ubiquitin C* (*UBC*) genes^9^. After processed to individual ubiquitins from their primary polyubiquitin precursor proteins, they are then transiently attaches to thousands of proteins either as a single molecule (monoubiquitination, mono-Ub)^10^ or as chains (polyubiquitination, poly-Ub)^11^. Since ubiquitin plays a key role in essential intracellular functions, the most important function of ubiquitin is believed to be regulation of protein turnover through the UPS.

Radiotherapy plays a critical role in curative management for the inoperable NSCLC patients^12^. Upon irradiation, numerous genes and proteins are involved in shifting the balance between cell survival and death^13^. Cancer cells may acquire resistance to radiation-induced apoptosis by dynamic interplay and regulation of multiple pro-survival factors^14^, which is considered as the major factor that impairs the outcomes of lung cancer patients^15^. Ubiquitin also plays an important role in DNA damage and repair response^16^,^17^. Recent studies have shown that both monoubiquitin and polyubiquitin modifications have critical roles in the DNA damage response (DDR), including the direct controlling protein expression levels^18^,^19^. Nuclear factor-κB (NF-κB), an import transcriptional factor, is a typical intrinsic cellular suppressor of apoptosis. After exposure to both low and high doses of ionizing radiation (IR), DNA damage-induced NF-κB promotes the expression of anti-apoptotic genes^20^,^21^. Therefore, the activated NF-κB has been reported to be a novel regulator of tumor radioresistance, and targeting NF-κB-mediated pathway is likely to contribute to radiosensitizer development^20^,^21^.

The effect of the whole cellular ubiquitin level on the proliferation and response to ionizing radiation remains to be further explored. Herein, we assess the expression level of ubiquitin in NSCLC tumor specimens and elucidate the effect of ubiquitin-knock down on the proliferation and radiosensitivity of lung cancer cells and its underlying mechanisms.

## Results

### Ubiquitin expression was significantly upregulated in human lung cancer tissues

**Figure 1:**
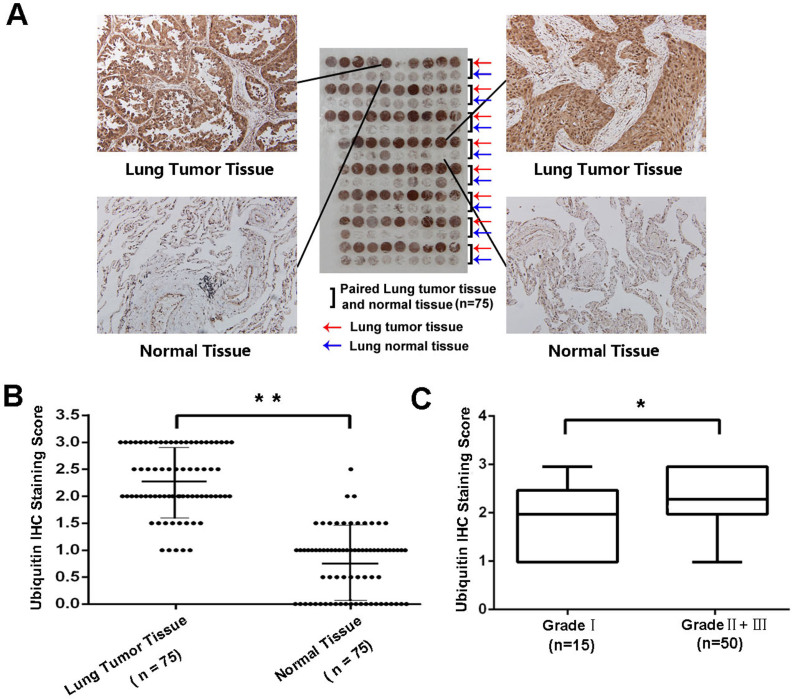
Immunohistochemical (IHC) staining of ubiquitin in lung cancer tissues and paired non-tumor tissue samples. (A) Representative images of the different staining patterns are presented. (B) Box plot depicting ubiquitin levels as assessed by IHC in the normal tissue and our series of 75 paired lung tumor cancer samples.(C) Ubiquitin level classified by according tumor histological features (Grade I, n = 15; Grade II+ III, n = 50). * *P* < 0.05, ** *P* < 0.01.

To determine the role of ubiquitin in lung cancer development, we first investigated the expression of ubiquitin in 75 paired clinical NSCLC specimens. The demographic information was summarized in Supplementary Table 1. The expression of endogenous ubiquitn between human lung cancer tissues and paired adjacent non-tumor tissues was analyzed by IHC staining. As shown in Fig. 1A, results showed that ubiquitin was expressed in both the nuclei and the cytoplasm of lung cancer cells. Among the 75 cases, positive ubiquitin expression was observed in 82.7% (62/75) of tumor tissues compared with 4.0% (3/75) of the paired adjacent non-tumor tissue (*P* < 0.0001, Fig. 1B and Table 1). The average ubiquitin staining score in tumor tissues (2.26 ± 0.60) was significantly higher than that in the paired adjacent normal tissues (0.75 ± 0.61). Moreover, stage II-III tumors showed significantly increased expression of ubiquitin compared with stage I tumor samples (Fig. 1C). Altogether, these data indicated that ubiquitin was overexpressed in lung cancer, and its overexpression may be associated with the progression of this malignancy.

**Figure 2:**
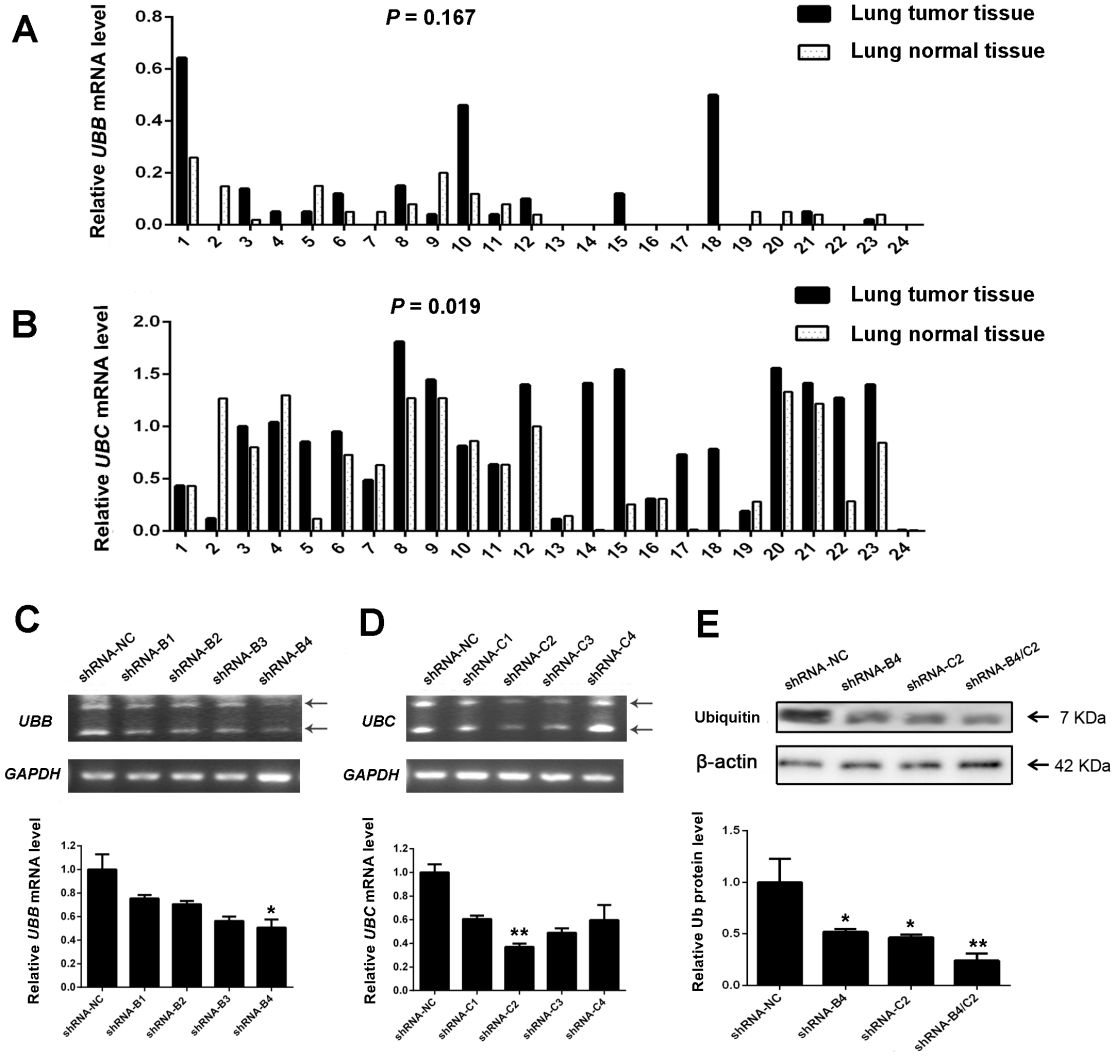
RT-PCR analysis of *UBB* and *UBC* mRNA in clinical NSCLC samples and the shRNA targeting ubiquitin silencing. RT-PCR was used to detect the expression level of *UBB* (A) and *UBC* (B) mRNA in 24 paired lung tumor and normal tissues. RT-PCR analyzed the silencing efficiency of (C) four shRNA targeting *UBB* gene (shRNA-*UBB*) and (D) four shRNA targeting *UBC* gene (shRNA-*UBC*). Arrows indicate the amplification product of tandem repeat sequence of ubiquitin coding genes. (E) Western blot was used to detect the expression of ubiquitin protein level after transfection of indicated vectors. * *P* < 0.05, ** *P* < 0.01.

To illustrate whether the increased ubiquitin level is attributed to *UBB* or/and *UBC* mRNA level, we performed reverse transcriptase-PCR (RT-PCR) to analyze the expression of *UBB* and *UBC* genes in 24 paired NSCLC tissues and corresponding normal tissues. As shown in Fig. 2A, 2B and Supplementary Fig. 1, results revealed that the expression of *UBC* gene remarkably increased in lung tumor tissues (*P* = 0.019), while *UBB* mRNA did not show statistical significance between the two groups (*P* = 0.167). The above results suggested that the increased ubiquitin in lung cancer tissues is likely to be ascribed to *UBC* transcripts.

### Knock-down of ubiquitin inhibited the proliferation of NSCLC *in vitro*

Ubiquitin mRNA and protein expression level in human lung cancer cell lines (H1299, A549, H460 and H1975) and normal bronchial epithelial cell lines (BEAS-2B and HBE) were analyzed by RT-PCR and Western blot. Results showed that both *UBB* and *UBC* transcripts and ubiquitin protein levels were generally more pronounced in human lung cancer cell lines (Supplementary Fig. 2A and 2B). Among the lung cancer cells, H1299 cells exhibited the highest ubiquitin level, possibly due to the *UBB* transcription. Moreover, the expression of ubiquitin in H1299 cells could be further induced by X-ray irradiation. (Supplementary Fig. 2C and 2D).

We next investigate the biological consequences of specific knock-down of ubiquitin in the NSCLC H1299 cells. Four different shRNA vectors were designed to silence the coding gene of *UBB* and *UBC*. After transfection with shRNA-NC, shRNA-*UBB*, shRNA-*UBC* or shRNA-*UBB/UBC* for 48 h, RT-PCR was performed to determine their silencing efficacy. The results revealed that shRNA-*UBB*-4 (shRNA-B4) targeting *UBB* gene and shRNA-*UBC*-2 (shRNA-C2) targeting *UBC* gene showed respective strongest inhibitory effect in H1299 cells (Fig. 2C and 2D). Western blot further confirmed that mixed transfection of shRNA-B4 and shRNA-C2 (shRNA-B4/C2) showed 76% inhibition of ubiquitin expression, compared with shRNA-NC transfected cells (Fig. 2E). Hence, shRNA-B4, shRNA-C2 and a combination of both was selected to perform the following experiments.

**Figure 3:**
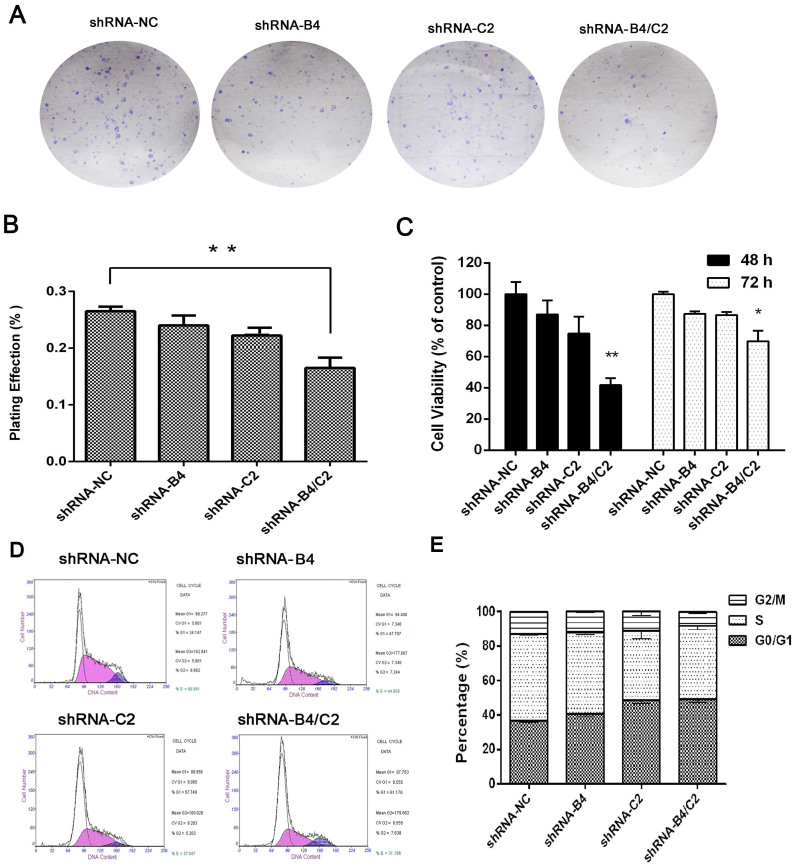
knock-down of ubiquitin affected the proliferation of H1299 cells. (A) Representative colony images. (B) Calculated relative colony formation rate. (C) The effect of ubiquitin silencing on cell viability of H1299 cells by using MTT assay and the shRNA-NC-transfected cells were normalized as 100%. (D) Representative charts for cell cycle distribution in ubiquitin knock-down and the control cells. (E) Calculated cell cycle distribution of in ubiquitin knock-down and the control cells. Data are expressed as means ± SEM from 3 separate experiments. * *P* < 0.05, **P < 0.01, compared with shRNA-NC group.

Then we evaluated the effect of ubiquitin silencing on the proliferation and colony formation of H1299 cells. Results revealed that ubiquitin downregulation generated less and smaller plate colonies (Fig. 3A and 3B). Cell growth monitored by MTT assay showed that knock-down of ubiquitin by shRNA-B4/C2 exhibited 32.9% reduction of cell viability compared with control shRNA-transfected cells (Fig. 3C), while treatment with either shRNA-B4 or shRNA-C2 had modest effects. Importantly, flow cytometry demonstrated that the percentage of cells in S phase was significantly decreased in ubiquitin knock-down cells, and the population of cells in G0/G1 phase was increased (Fig. 3D and 3E). Taken together, these results indicated that knock-down of ubiquitin remarkably inhibited cell growth by modulating the cell cycle in H1299 cells.

### Knock-down of ubiquitin increased the radiosensitivity in H1299 cells

**Figure 4:**
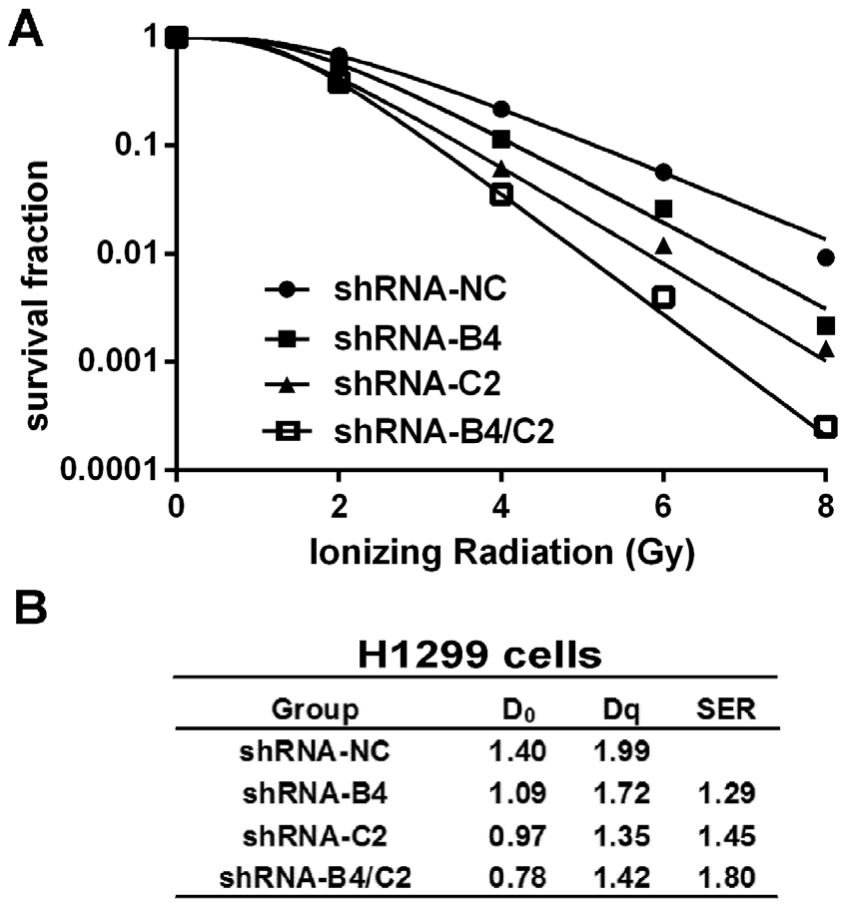
Clonogenic survival rate of H1299 cells after knock-down of ubiquitin treatment with X-ray irradiation. (A) Clonogenic cell survival curves were generated for H1299 cells that were treated with shRNA for 24 h and then were exposed to 2, 4, 6 or 8 Gy of X-ray irradiation. The survival data were normalized to those of the unirradiated control cells. (B) The D_0_, D_q_ and SER value in control cells and ubiquitin knock-down cells. The SER was calculated according to the multi-target single hit model.

We next performed clonogenic survival assays to investigate the impact of ubiquitin on radiosensitivity in lung cancer H1299 cells. Cells were transfected with shRNA-NC, shRNA-B4, shRNA-C2 or shRNA-B4/C2 24 h prior to irradiation at 0, 2, 4, 6 and 8 Gy. The main parameters of H1299 cells in dose-survival curves were obtained according to the multi-target single hit model. A dose-dependent radiosensitization on ubquitin-silencing was also observed with a sensitizing enhancement ratio (SER) of 1.29 and 1.45 by shRNA-B4 and shRNA-C2, respectively (Fig. 4A). This moderate radiosensitizing effect might partially due to a decrease of cellular ubiquitin level. Transient ubiquitin knock-down (shRNA-B4/C2) gave rise to a greater radiosensitization. The mean lethal dose (D_0_) of the radiation alone group and irradiation plus ubiquitin knock-down (shRNA-B4/C2) group were 1.40 and 0.78 Gy, while the quasi-threshold dose (Dq) were 1.99 Gy and 1.42 Gy, with SER value increasing to 1.80 (Fig. 4B). Thus, downregulation of ubiquitin significantly sensitized H1299 cells to irradiation.

### Knock-down of ubiquitin increased radiation-induced apoptosis and DNA damage in H1299 cells

**Figure 5:**
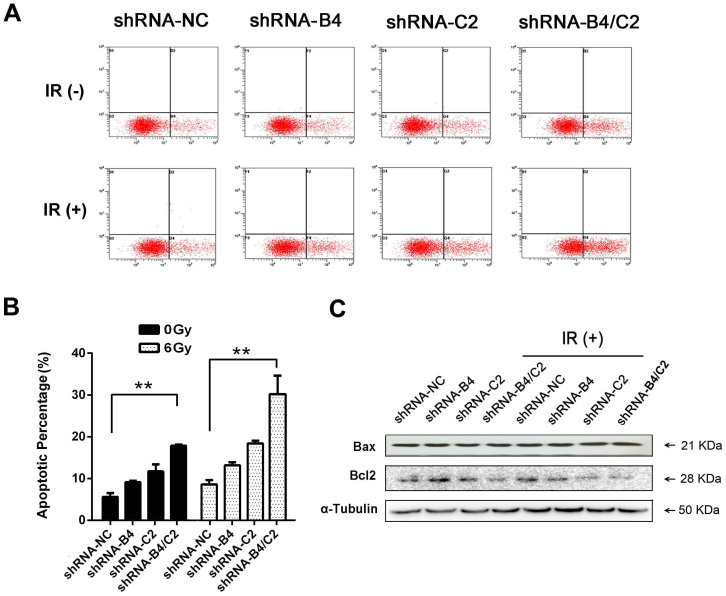
Silencing of ubiquitin enhanced the radiation-induced apoptosis. H1299 cells were transfected with shRNA for 24 h prior to 6 Gy irradiation. (A) Apoptosis were measured using Annexin-V/PI staining in H1299 cells. (B) Quantification of apoptosis percentage. (C) Western blot detected the expression of apoptosis related proteins Bcl-2 and Bax. ** *P* < 0.01.

We then assessed the apoptosis of H1299 cells after knock-down of ubiquitin and/or radiation. The proportion of apoptotic cells was determined by flow cytometry after irradiation. Compared with cells treated with radiation alone, the group in which ubiquitin was knocked down combined with X ray irradiation had a clear increase of apoptosis percentage (Fig. 5A and 5B). As shown in Fig. 5C, knock-down of ubiquitin downregulated the expression of anti-apoptotic protein Bcl-2, but did not upregulate the expression of pro-apoptotic protein Bax in our study.

**Figure 6:**
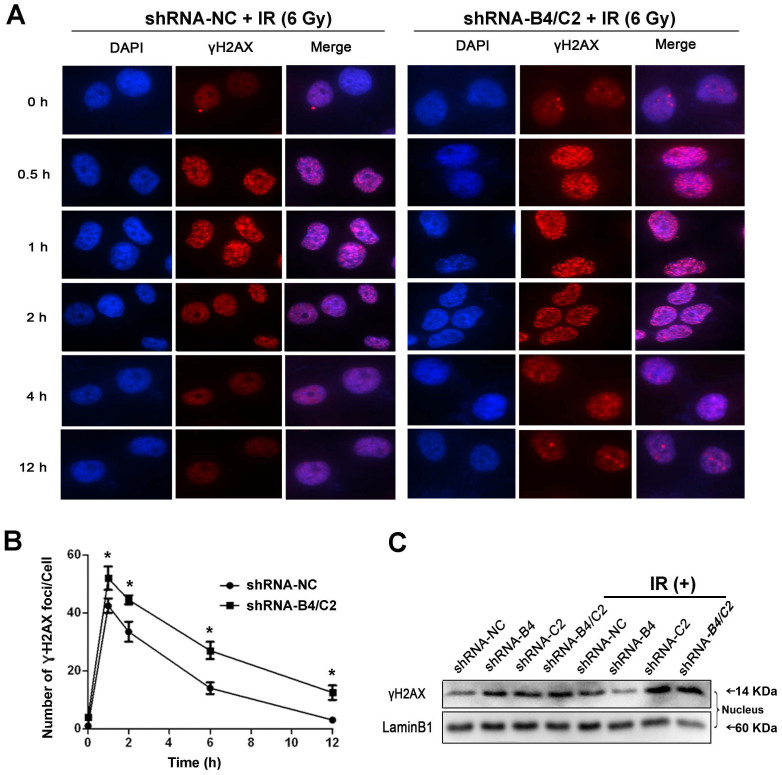
Ubiquitin downregulation increased γH2AX foci induced by X-ray irradiation. H1299 cells were transfected with indicated shRNAs and then sham irradiated or irradiated with 6 Gy X-ray. (A) Immunofluorescence of γH2AX foci (red) in H1299 cells for an additional 0, 0.5, 1, 2, 4 or 12 h. Nuclei were stained with DAPI (blue). Images were acquired using a fluorescent microscope. (B) Kinetics of γH2AX foci loss in different groups of cells after 6 Gy X-ray irradiation. The mean number of γH2AX foci per cell (foci/cell) was counted. * *P* < 0.05, compared with shRNA-NC group. (C) Western blot was performed to detect the γH2AX foci induced by X-ray irradiation after transfection with indicated shRNA vectors.

Immunofluorescence was subsequently used to examine the formation of γ-H2AX foci in the nuclei. The results revealed that the formation of γ-H2AX foci after 6 Gy X-ray irradiation was significantly more pronounced by knock-down of ubiquitin (Fig. 6A and 6B). Western blot analysis confirmed that knock-down of ubiquitin inhibited the clearance of nuclear γ-H2AX foci induced by irradiation (Fig. 6C), which is consistent with the results from immunofluorescence staining. Meanwhile, radiation plus ubiquitin knock-down showed no change in cell cycle distribution, indicative the irrelevance of cell cycle distribution (Supplementary Fig. 3). Taken together, these results revealed that radiation obviously increased apoptosis fraction and DNA damage.

### Knock-down of ubiquitin decreased p-AKT and inhibited NF-κB translocation into the nucleus after exposed to X-ray irraddiation

**Figure 7:**
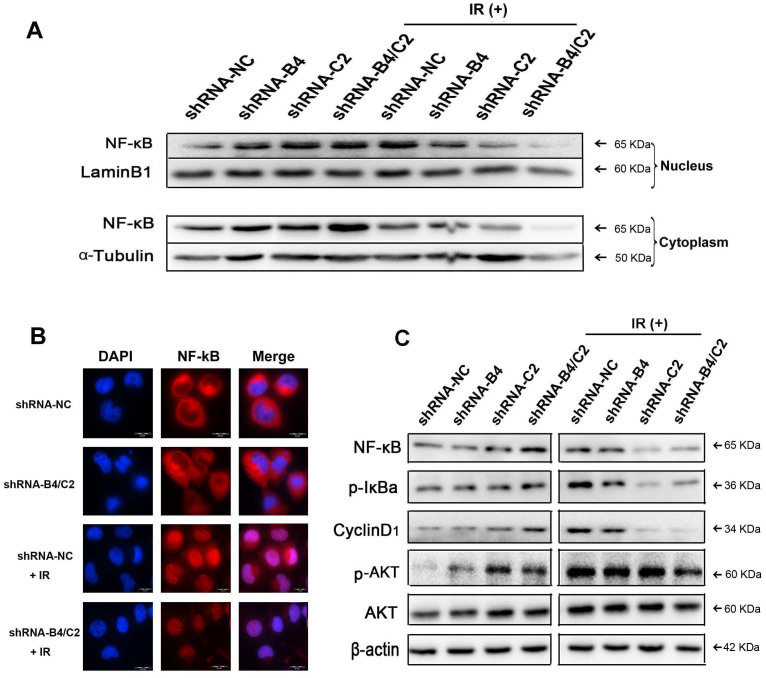
Knock-down of ubiquitin affected AKT activation and inhibited NF-κB translocate induced by X-ray irradiation. H1299 cells were transfected with indicated shRNA for 24 h prior to sham irradiation or 6 Gy X-ray irradiation. (A) Western blot showing the nuclear and cytoplasmic distribution of NF-κB with sham irradiation or 6 Gy X-ray irradiation in H1299 cells. (B) Immunofluorescence analysis of NF-κB distribution in nucleus and cytoplasm of H1299 cells. (C)The expression levels of NF-κB, AKT, p-AKT, p-IκBα, Cyclin D1 and the internal control β-actin were measured by Western blot. The samples were derived from the same experiment and were run under the same experimental conditions. The uncropped blots details are provided in Supplementary Information.

NF-κB has been extensively reported to be implicated in cell radiosensitivity due to their crucial role in radiation response. To further confirm the effect of ubiquitin knock-down on NF-κB activity induced by radiation, we analyzed the expression level and distribution of NF-κB (p65 subunit) in H1299 cells. As shown in Fig. 7A, Western blot analysis showed that the expression levels of p65 in the nuclear extracts of the H1299 cell lines were increased after both ubiquitin knock-down and irradiation, while knock-down of ubiquitin decreased radiation-induced NF-κB (p65) expression in both nucleus and cytoplasm. Result from immunofluorescence also revealed that irradiation exposure induced the translocation of the p65 protein from the cytoplasm into the nucleus in H1299 cells, which was attenuated by ubiquitin knock-down (Fig. 7B). Meanwhile, phosphorylation of IκBα, which mediates the degradation of IkBα and activates NF-κB, was decreased, suggesting that decreased NF-κB expression was attributed to decreased degradation of IκBα (Fig. 7C). Moreover, Western blot also revealed that irradiation plus ubiquitin inhibition decreased Cyclin D1 and phospho-AKT expression (Fig. 7C), suggesting that AKT and Cyclin D1 pathways are also involved in ubiquitin-modulated radiosensitivity. Our results also indicated that knock-down of *UBB* and *UBC* gene progressive increased the expression of phospho-AKT, NF-κB and Cyclin D1, and a combination of the both showed the most enhancement.

### Knock-down of ubiquitin inhibited tumor cell growth and enhanced radiosensitivity in a mouse xenograft model

**Figure 8:**
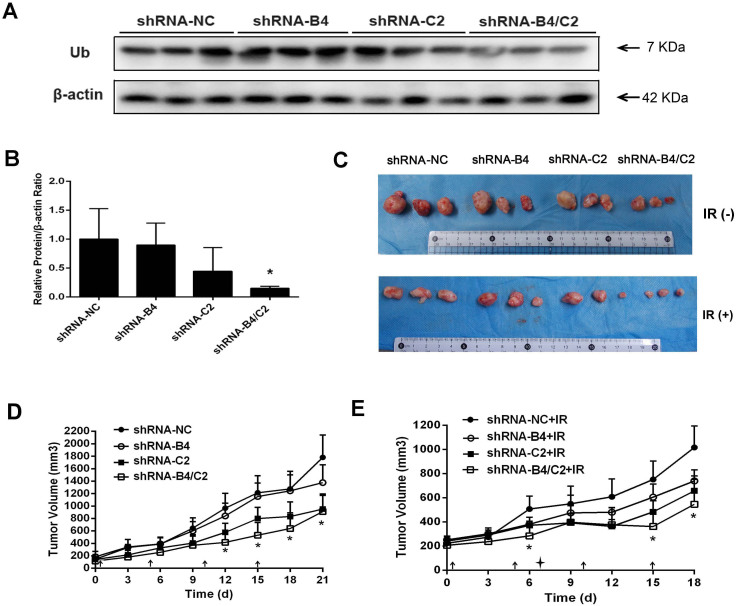
Knock-down of ubiquitin and/or plus X-ray irradiation affected the growth of xenografts tumor. Each group of mice was composed of five male nude mice. H1299 cells were inoculated into the right posterior flank region of BALB/c nude mice. (A) Verification the silencing effect of the shRNA plasmid transfected *in vivo*. Western blot showing the silencing of ubiquitin in each group (shRNA-NC, shRNA-B4, shRNA-C2 and shRNA-B4/C2). (B) Relative expression of ubiquitin of each group, * *P* < 0.05. (C) Representative tumors from the mice of each group at the end time-point. (D) Tumor volume growth curve of various ubiquitin knock down and (E) combined ubiquitin knock down with irradiation by animal assay. The tumor size was measured at a 3 day intervals. (arrows represent intratumorally injection of shRNA; starlike symbol means treatment with 8 Gy of X-ray irradiation).

For the purpose of examining the inhibitory effect of ubiquitin knock-down on tumor cell growth *in vivo*, H1299 cells were inoculated subcutaneously into the right posterior flank region of BALB/c nude mice. When tumors reached 150–200 mm^3^ in volume, the mice were randomly divided to 4 groups. Either shRNA control (shRNA-NC) or shRNA targeting ubiquitin coding genes was injected intratumorally to the xenografts of nude mice once every five days for four times (Fig. 8D, indicated by the arrows). Mice in each group showed no difference in body weight and no gross pathologic abnormality, indicating no observable toxicity (Supplementary Fig. 4). As shown in Fig. 8A and 8B, transfection of shRNA-B4/C2 significantly decreased the ubiquitin level of transplantation tumors, compared with the control group (85% reduction, *P* < 0.05). In comparison, transfection with either shRNA-B4 or shRNA-C2 had modest inhibitory effect. Compared with shRNA-NC group, combined silencing of *UBB* and *UBC* (shRNA-B4/C2) inhibited 51.51% of tumor volume (Fig. 8C upper panel and Fig. 8D), which was more effectively than that of shRNA-B4-transfected group (27.12%) or shRNA-C2-transfected group (45.05%). Given that downregulation of the ubiquitin level of H1299 cells decreased the tumor proliferation, the 50% reduction of tumor size is likely to be attributed to the effect of ubiquitin depletion.

The effect of combined treatment with X-ray irradiation plus ubiquitin silencing on the growth of H1299 xenografts was also determined. As shown in Fig. 8E, there is no significant difference in tumor volume between groups before treatment. The tumor volume of mice treated with radiation alone was reduced by 41.22% compared with the control group. Comparatively, H1299 xenografts that received combined treatment of 8 Gy X-ray irradiation and ubiquitin knock-down exhibited much smaller tumors with a volume reduction of 79.10%, compared with mice that received radiation alone (*P* < 0.05, Fig. 8C lower pannel and 8E, indicated by starlike symbol).

### Inhibition of ubiquitin decreased the proliferation and enhanced apoptosis in response to X-ray irradiation in vivo

**Figure 9:**
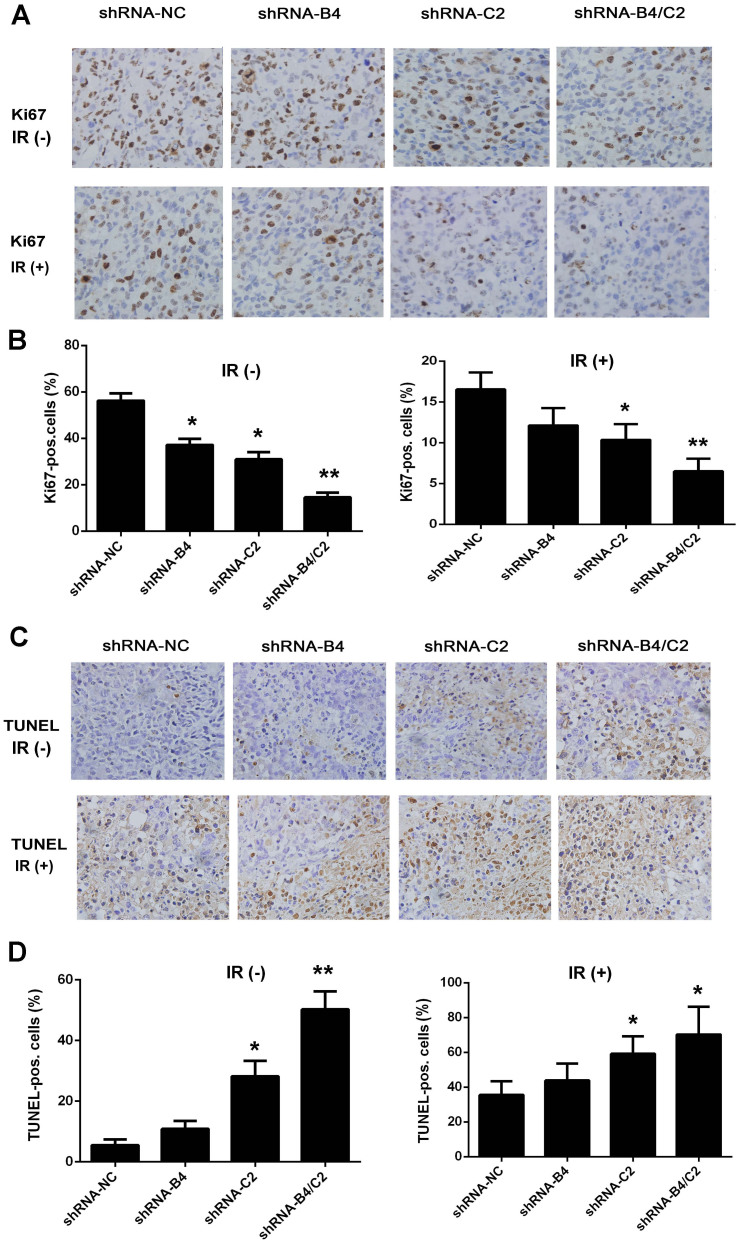
Ubiquitin silencing affect the proliferation and apoptosis response to X-ray irradiation *in vivo*. (A) Representative image of IHC staining of Ki67 expression in each group of the H1299 xenografts. (B) Quantification of Ki67 positive cells (%) in each field. (C) Representative image of IHC staining of TUNEL and (D) TUNEL positive cells (%) in each group. All brown objective bodies in tumor mouse samples were counted using an Olympus DP70 microscope. Figures are photographed at a 200× magnification. The column diagrams showed the average percentage of Ki67 positive and TUNEL-positive cells of 10 random fields. * *P* < 0.05, ** *P* < 0.01, compared with shRNA-NC group or shRNA-NC plus 8 Gy X-ray irradiation.

To characterize the molecular changes in the ubiquitin silenced xenografts, proliferation marker Ki67 and cell apoptosis were determined. As shown in Fig. 9A and 9B, Ki67 positive cells were significantly decreased by 41.60% in H1299 tumors in the combined knock-down of *UBB* and *UBC* gene group (*P* < 0.01). When treated with ubiquitin silencing plus irradiation, the Ki67 positive cells in the xenografts were significantly reduced by 10.03% (*P* < 0.01), compared with shRNA-NC plus irradiation group. We further validated TUNEL positive cells in the nude mice xenografts treated with ubiquitin silencing and/or irradiation. TUNEL positive cells were significantly increased by 44.80% (*P* < 0.01) after treatment with the combinational knock-down of *UBB* and *UBC* gene, while traetment with ubiquitin silencing and irradiation significantly increased the TUNEL-positive cells by 34.86% (*P* < 0.05; Fig. 9C and 9D). These data indicate that combined knock-down of ubiquitin coding genes *UBB* and *UBC* reduced Ki67 expression and improved its antitumor effect *in vivo*. Moreover, ubiquitin silencing could enhance the radiosensitivity of H1299 xenografts, which is consistent with the results from *in vitro* study.

## Discussion

The UPS is conservative in all eukaryotes and plays key roles in the regulation of numerous cellular functions. Accumulating evidence shows that elevated level of ubiquitin has been observed in multiple types of cancers^22^,^23^,^24^,^25^,^26^. Nevertheless, few study illustrated the influence of ubiquitin on cellular function of cancer cells. This study, for the first time to our knowledge, demonstrates the upregulation of ubiquitin in NSCLC tissues. The increased ubiquitin in lung cancer tissues is likely to be ascribed to *UBC* transcripts. We also showed that knock-down of *UBB* and *UBC* that coding ubiquitin resulted in dramatic growth inhibition and radiosensitivity enhancement in NSCLC cells. Moreover, we provide molecular evidences that ubiquitin knock-down weakened the resistance of lung cancer cells to X-ray irradiation through apoptosis, DNA damage repair and NF-κB signaling pathways.

In most cancers, tumor cells acquire their malignant capabilities primarily due to their high metabolic state and speedy material metabolism^27^. UPS is involved in busy carrying cellular garbage disposal for recycling. Moreover, cancer cells acquire their malignancy through a number of mutations and extensively reprogrammed pathways, which inevitably generate a variety of cellular stresses^28^,^29^. Thus, cancer cells must tolerate these increased stresses through stress-supporting systems such as the heat shock response (HSR) and the ubiquitin system to survive^30^. The elevated level of ubiquitin in cancer cells is believed to contribute to the reinforcement of the high metabolic and stress-support system through ubiquitination^30^,^31^. Since the approval of clinical use of bortezomib in 2003 by FDA, there have been substantial progresses in developing potential anticancer drugs that target various key molecules in ubiquitin-proteasome pathway^32^. Deubiquitinating enzyme inhibitors and inhibitors of E1, E2 and E3 ligands involved in UPS have been now in clinical or preclinical trial^33^,^34^,^35^,^36^. The 76-amino acid protein tag ubiquitin affects cellular process by regulating protein degradation, activating and inactivating proteins, and modulating protein-protein interactions by the addition of a single ubiquitin molecule (monoubiquitination) or different types of ubiquitin chains (polyubiquitination). Inhibitors or activators of ubiquitin conjugation or deconjugation have been proved to be attractive cancer therapeutic targets^37^. Our study shows that ubiquitin itself can also be used as a novel lung cancer target, which supports the critical role of UPS in cancer progression and treatment.

In our study, inhibition of *UBB* and *UBC* could dramatically enhance the phosphorylation of AKT (Fig. 7C) and increase the expression of total NF-κB (p65, Fig. 7C). Moreover, ubiquitin silencing also promoted the translocation of p65 into nucleus. These results are consistent with previous report that *UBB* knock-down enhances p65 expression in both cytoplasm and nucleus of Hela cells^30^. Proteasome inhibitor MG132 has been reported to stabilize IκBα^38^, which is required for the activation of NF-κB and its translocation. Therefore, the increased NF-κB protein level and nuclear localization by ubiquitin knock-down may be ascribed to the stabilization of IκBα by UPS inhibition. Moreover, the degradation of NF-κB itsself may also be attenuated due to lack of ubiquitin.

Numerous studies have shown that ubiquitin is involved not only in protein degradation, but also in protein modification^39^,^40^. Upon irradiation, multiple molecules are activated by ubiquitination and initiate DNA repair^6^,^16^. For example, neither monoubiquitinylation nor polyubiquitinylation of PCNA affects its degradation, whereas blockade of K164 PCNA residue that is normally ubiquitinylated inhibits DNA repair^41^. We show that silencing ubiquitin significantly increased the number of γH2AX, indicative of inhibited DNA repair of H1299 cells exposed to irradiation. NF-κB is a well-established regulator of cell survival, transformation, differentiation, invasion and radiation response^42^,^43^. By inhibiting the proteasome, bortezomib redistributes the cell cycle and inhibits the transcriptional capability of NF-κB, which modulates cell proliferation and apoptosis^44^,^45^. Our results suggest that downregulation of ubiquitin decreased the expression of NF-κB and inhibited its translocation to the nucleus by decreased phospho-IκBα after irradiation, which may attenuated NF-κB-mediated radiation response^20^,^21^. Upon irradiation, ubiquitin silencing may suppress cellular response to radiation-induced DNA damage rather than protein degradation. Therefore, the effect of ubiquitin silencing on NF-κB (p65) expression with and without irradiation may be different. The underlying mechanism warrants in-depth exploration.

Our results revealed that combined inhibition of *UBB* and *UBC* was similar to the effect of single *UBC* silencing (Fig. 7C), indicating that *UBC* gene represented a more effective radiosensitizing target. Cyclin D1, a NF-κB downstream effector, was inhibited after ubiquitin knock-down. The expression of Cyclin D1 has been implicated in cell survival^46^. Other important mechanisms associated with cancer cell radiosensitivity include apoptosis and cell cycle arrest. Both our *in vitro* and *in vivo* studies suggest that a reduced level of ubiquitin is essential to allow H1299 cells to promote apoptosis, which is similar to a previous report of *UBB*-knock-down in hepatoma and cervical cancer cells^30^,^47^. In contrast, targeting ubiquitin showed no significant effect on cell cycle distribution after irradiation in our study, which was in discrepancy with proteasome inhibitors that modulating cell cycle arrest^48^,^49^. Whether other mechanisms are involved in the enhancement of radiosensitivity of *UBB* and *UBC* gene in lung cancer cells also warrants further investigation.

Taken together, our results demonstrate that ubiquitin is overexpressed in NSCLC and that downregulation of ubiquitin inhibits the proliferation and enhances the efficacy of radiotherapy via multiple pathways in NSCLC cells both *in vitro* and *in vivo*. Targeting ubiquitin may serve as a potentially important and novel approach for human NSCLC prevention and therapy.

## Methods

### Clinical specimens

Fresh lung cancer tissues were collected between 2010 and 2012 from Suzhou Municipal Hospital (Suzhou, China) and Changzhou Tumor Hospital affiliated to Medical School of Soochow University (Changzhou, China). All patients were diagnosed with histologically confirmed NSCLC, and the histological features of the specimens were evaluated by two senior pathologists according to the classification criteria from the World Health Organization (WHO)^50^. Signed informed consent for sample collection was obtained from all patients. Each lung cancer tissue sample has matched adjacent non-tumor lung tissue removed during the same surgery. For PCR analysis, specimens were snap frozen in liquid nitrogen and then stored at −80°C until use. For immunohistochemical analysis, samples were fixed with formalin and embedded with paraffin. These tissue samples were then processed into tissue arrays by Suzhou Xinxing Biotech (Suzhou, China). All experiments were performed in accordance with the relevant guidelines and regulations of Soochow University. And this study was approved by the Ethical Committee of Soochow University.

### Immunohistochemical staining

Immunohistochemical (IHC) staining were performed to detect the ubiquitin expression level in tissues from lung cancer patients and samples from H1299 xenografts of nude mice were used for IHC staining of Ki67. The paraffin-embedded sections were stained with antibodies against ubiquitin or Ki67 antibody (both from Santa Cruz Biotechnology, Santa Cruz, CA) for 1 h at dilution 1:1000 as the previously reported method^51^. Ubiquitin IHC staining was based on the proportion of cell staining and scored from 0 to 3 criteria as described previously^52^. An overall score of <2 was defined as negative, while a score ≥ 2 was defined as positive. The slides were analyzed by standard light microscopy. Negative controls were stained with IgG as primary antibody.

### Reverse transcriptase-PCR

Total RNA was extracted using TRIzol reagent (Tiangen, Beijing, China). RNA was reverse transcribed using a reverse transcription kit (Thermo-Fisher, Waltham, MA) under 42°C for 60 min and 70°C for 5 min. The expression level of *UBB* and *UBC* gene in each sample was measured by RT-PCR. *GAPDH* was used as an endogenous standard. The primer sequences were listed in Supplementary Table 3.

### Cell cultures and irradiation

The human normal bronchial epithelial cell lines (BEAS-2B and HBE) and the human lung cancer cell lines (H1299, A549, H460 and H1975) were maintained in DMEM supplemented with 10% FBS and antibiotics (100 units/ml penicillin G, 100 units/ml streptomycin sulfate; Gibco, Grand Island, NY) at 37°C in 5% CO2. Cells were exposed to a single dose of X-rays using the linear accelerator (RadSource, Suwanee, GA) at a dose rate of 1.15 Gy/min.

### shRNA construction and transient transfection

Short hairpin RNAs (shRNAs) targeting ubiquitin coding genes *UBB* and *UBC* were designed and constructed by GenePharma Co. Ltd. (Shanghai, China). The shRNA sequences and locations were listed in Supplementary Table 2. Cells were transfected with constructed vectors by Lipofectamine 2000 (Invitrogen, Calsbad, CA).

### Cell proliferation assays

The effect of ubiquitin on cell viability was monitored by the MTT colorimetric assay. In brief, after an overnight incubation, H1299 cells (2 × 10^3^ cells/well) were transfected with shRNA-*UBB*, shRNA-*UBC* or shRNA-*UBB/UBC* mixture (with the same total quantity) in 96-well plates, and were incubated for another 24 or 48 h. Then the cells were incubated with 10 μL of 5 mg/mL MTT for 5 h. Then, the supernatant was removed and each well was added with 150 μl of dimethylsulfoxide (DMSO) to dissolve the formazan for 10 minutes by vibration. The optical density was measured at 492 nm with a microplate reader (Bio-Rad, Hercules, CA). The viability index was calculated as the experimental OD value/the control OD value (which were arbitrarily assigned as a viability of 100%). Three independent experiments were performed in quadruplicate.

### Cell cycle progression analysis

The distribution of H1299 cell cycle was assessed by flow cytometry 24 h after shRNA transfection. Cells were harvested by trypsin digestion, pelleted by centrifugation, washed with ice-cold PBS, then fixed with 70% cold ethanol overnight. The staining solution containing propidium iodide (50 μg/ml) (Sigma-Aldrich, St. Louis, MO) and DNAse-free RNAse (20 μg/ml) were added 30 min before detection. The fraction of the population in each phase of the cell cycle was determined as a function of the DNA content using a coulter flow cytometry (Beckman-Coulter, Brea, CA).

### Clonogenic assay

For standard clonogenic assays, the cells were treated with shRNA transfection for 5 h. and then cells were re-suspended and seeded into six-well plates at 200–6,000 cells/well depending on the dose of radiation. The cells were irradiated with 0, 2, 4, 6 or 8 Gy X-ray irradiation by linear accelerators at a dose rate of 1.15 Gy/min. After the irradiation, the cells were grown from 7–10 days to allow for colony formation and were subsequently fixed and stained using crystal violet. Colonies consisting of 50 or more cells were counted as a clone. The radiation sensitivity enhancement ratio (SER) was measured according to the multi-target single hit model.

### Apoptosis analysis

Cells were transfected with shRNA 24 h prior to treatment with sham or 6 Gy X-ray irradiation. Apoptosis was measured using propidium iodide (PI)/Annexin-V double staining following manufacturer's instructions (BD Biosciences, San Jose, CA). Cells were harvested 48 h after irradiation and apoptotic fractions were measured using flow cytometry (Beckman, USA). The Annexin-V+/PI- cells are early in the apoptotic process, the Annexin-V+/PI + cells indicating late apoptosis. The percentage of both kinds of cells was counted.

The terminal deoxynucleotidyl transferase dUTP nick-end labeling (TUNEL) assay was performed to detect the apoptosis of xenograft tissue samples. 5 μm sections from H1299 xenografts were immersed in xylene and hydrated in decreasing concentrations of ethanol, and the TUNEL assay was performed following manufacturer's instructions (Keygen, Nanjing, China). Ten random fields from 3 slides per group were counted. TUNEL-positive brown nuclei within tissues were counted. Data were expressed as the percentage of apoptotic cells per field.

### Protein extraction and Western blot

H1299 cells (1 × 10^5^ cells/dish) in 6-well plates were grown in DMEM containing10% FBS to a 70% confluent. The cells were trasfected with shRNA for 24 h and then with sham or 6 Gy X-ray irradiation. Cytosolic and nuclear protein was extracted 24 h after irradiation using the cytosolic and nuclear protein extract kit (Beyotime, Nantong, China). In brief, cells were harvested in lysis buffer with 1 mM phenylmethyl-sulfonyl fluoride (PMSF). The cells were allowed to swell on ice for 15 min. The homogenates were centrifuged for 5 min at 12,000 × g and the supernatant was used as the cytosolic extract. The nuclear pellet was re-suspended in cold extraction buffer and the samples were centrifuged at 15,000 × g for 30 min. The obtained supernatant was used as the nuclear extract.

Proteins were separated by sodium dodecyl sulfatepoly-acrylamide gel electrophoresis (SDS-PAGE) through 10% gel and transferred onto PVDF membranes. The gels have been run and transferred under the same experimental conditions. The membranes were blocked with 5% non-fat dried milk in TBS containing 1% Tween-20 (TBST) for 2 h at the room temperature, followed by incubation with the appropriate Bax, Bcl-2, Cyclin D1 (Santa Cruz Biotechnology, Santa Cruz, CA), NF-κB, AKT, phospho-AKT and phospho-IκBα primary antibodies (Epitomics, Burlingame, CA) at a 1:2,00-1:1,0000 dilution for 2 h, and either horseradish peroxidase-conjugated goat anti-rabbit or anti-mouse antibodies for 1 h. β-actin, α-Tubulin and LaminB (all from Beyotime, Nantong, China) were served as the loading controls. Antibody-bound proteins were detected using an ECL Stable Peroxide solution (PointBio, Shanghai, China). All protein bands were visualized by a FluroChem MI imaging system (Alpha Innotech Santa Clara, CA) under the room temperature.

### Immunofluorescence

Cells were were transfected with shRNA 24 h before exposed to irradiation. After irradiation, the cells were fixed with 4% paraformaldehyde for 10 min, treated with 1% Triton X-100 for 10 min, and then incubated with blocking serum for 1 h at room temperature. After washing with PBS, samples were incubated with a rabbit monoclonal against γH2AX or NF-κB antibody (both from Epitomics, Burlingame, CA) over night at 4°C, followed by FITC-conjugated goat-anti-rabbit secondary antibody (1:200) for 2 h. Nuclei were counterstained with 4,6-diamidino-2-phenylindole (DAPI; Sigma-Aldrich, St. Louis, MO). The cells were observed using an Olympus fluorescence microscope (Olympus, Tokyo, Japan). γH2AX foci were counted in each cell. At least 100 cells were counted from 10 randomly chosen fields of view.

### Xenograft studies of nude mice

4-week-old BALB/c athymic nude mice were purchased from Shanghai SLAC Laboratory Animal Co., Ltd. (Shanghai, China). The mice were maintained under standard laboratory conditions on a 12-h light–dark cycle and given access to sterilized food and water ad libitum under specific pathogen-free environment. For the subcutaneous injection, H1299 cells (4 × 10^6^) were suspended in 100 μl PBS and then inoculated subcutaneously into the right posterior flank region of BALB/c nude mice. Each group consisted of five nude mice. Two-dimensional measurements were taken with an electronic caliper, and the tumor volume in mm^3^ was calculated using the formula: volume = a × b^2^ × 0.52^53^, where a is the longest diameter, b is the shortest diameter. When a tumor reached 150–200 mm^3^ in volume, the mice were random divided into 4 groups and injected intratumor respectively with shRNA control (shRNA-NC), shRNA-*UBB*, shRNA-*UBC* or shRNA-*UBB/UBC* together with transfection reagents. Tumors were irradiated by a tailor-made irradiator to a signal dose of 8 Gy X-rays from linear accelerators (Varian, Palo Alto, CA) at a dose rate of 200 cGy/min on day 7. At the terminal point when the mice were sacrificed, tumors were frozen in −80°C or fixed in 10% formalin overnight and subjected to routine histological examination.

Mice were observed for lethargy, appetite, and sacrificed timely before natural death occurred. The date on which the grossly visible tumor stabilized appeared for a volume of 150–200 mm^3^ was recorded and random distributed. Both the tumor size and animal weight were measured every 3 days. All of the animals were treated according to protocols approved by the Institutional Animal Care and Use Committee of Soochow University. And this study was approved by the Institutional Animal Care and Use Committee of Soochow University.

### Statistical data analyses

Data were expressed as the means ± SEM of at least three independent experiments. The data were then analyzed using Student's *t*-test when only two groups were present, or assessed by one-way analysis of variance (ANOVA) when more than two groups were compared. The interaction between ubiquitin knock-down and radiation was tested using two-way ANOVA. The sensitizer enhancement ratios (SER) were measured according to the multi-target single hit model. Expression difference of ubiquitin mRNA and protein between lung tumor tissues and the adjacent normal tissues were analyzed using paired *t*-test and Pearson Chi-square (χ^2^) test. Differences were considered statistically significant when *P* < 0.05. SPSS Statistical software (version 19.0) was utilized for statistical analysis.

## Author Contributions

S.Y.Z. and J.P.C. conceived and designed the study. Y.T.T., J.D.L. and Y.Y.G. carried out the molecular biology studies. Y.T.T. drafted the manuscript and the figures. C.C.M., W.H.S. and M.L. performed the animal experiment. W.Z. and X.F.Z. performed the statistical analysis. S.Y.Z. modified the manuscript. All authors read and approved the final manuscript.

## Supplementary Material

Supplementary Information Supplemental materials

## Figures and Tables

**Table 1:** The expression of ubiquitin in lung cancer tissues and adjacent normal tissues

		Total	Ubiquitin (−)	Ubiquitin (+)	*Chi-square*	*P* value
Lung cancer tissue		75	13 (17.3%)	62 (82.7%)	91.33	<0.0001
Non-tumor tissue		75	72 (96.0%)	3 (4.0%)		
