# Supplementary material for: Downregulation of ubiquitin inhibits the proliferation and radioresistance of non-small cell lung cancer cells in vitro and in vivo
Source: Sci Rep. 2015 Mar 30;5:9476. doi: 10.1038/srep09476 (PMC4377628; doi:10.1038/srep09476)
Supplement: Supplementary Information — Supplemental materials [file srep09476-s1.pdf]

Supplemental information

Title: Downregulation of ubiquitin inhibits the proliferation and radioresistance of non-small cell lung cancer cells *in vitro* and *in vivo*

By：Yiting Tang, Yangyang Geng, Judong Luo, Wenhao Shen, Wei Zhu, Cuicui Meng, Ming Li, Xifa Zhou, Shuyu Zhang, Jianping Cao

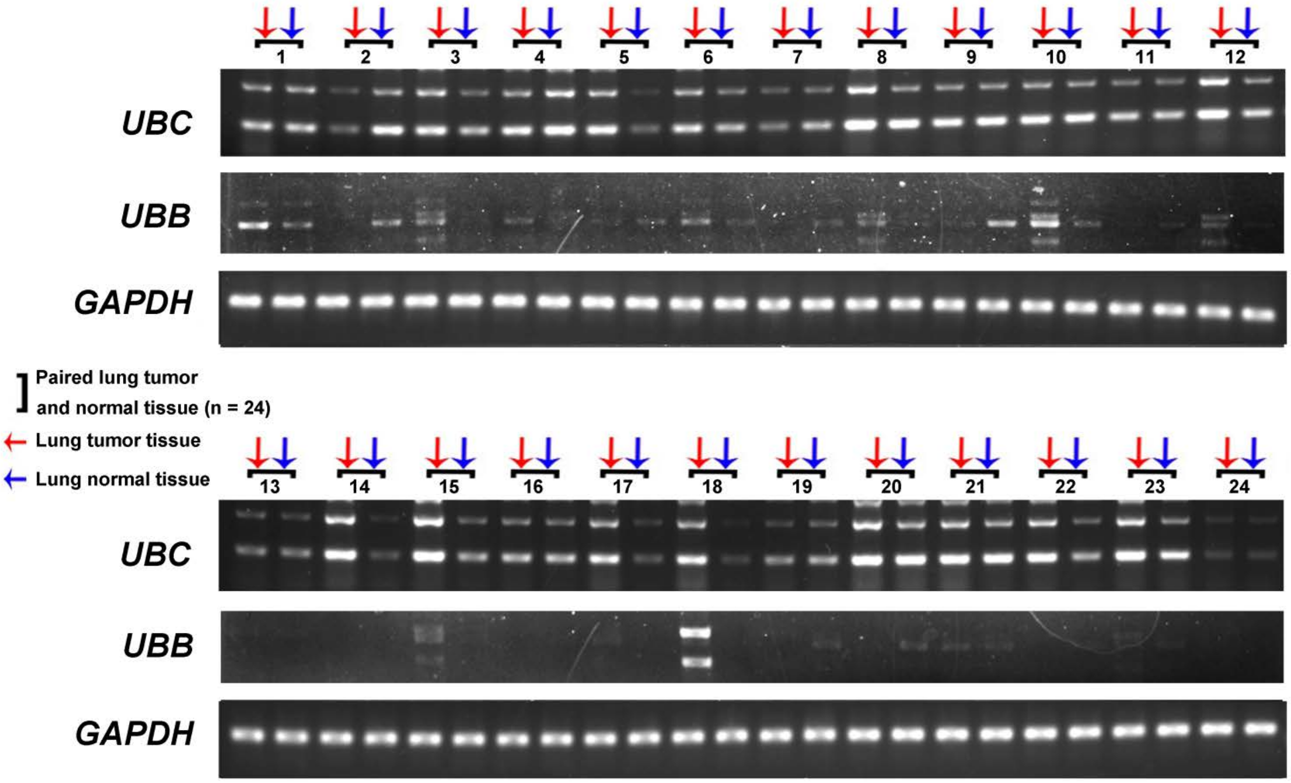

Supplemental Figure 1

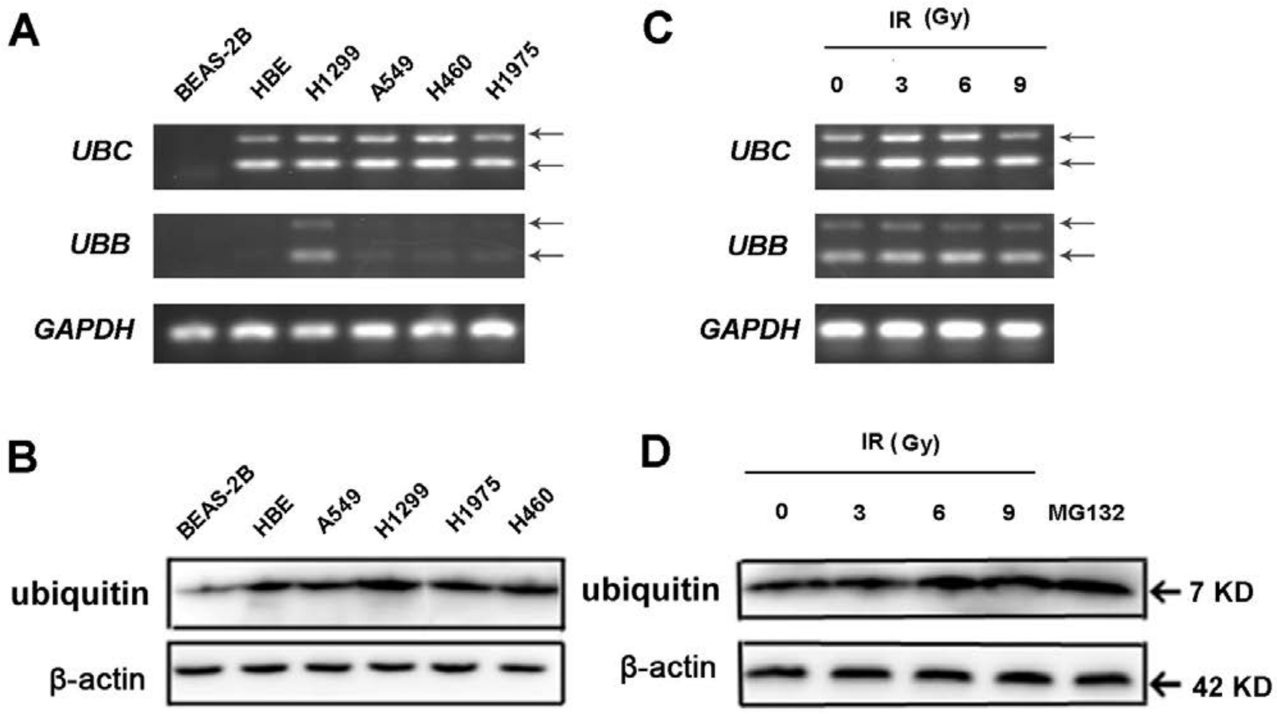

Supplemental Figure 2

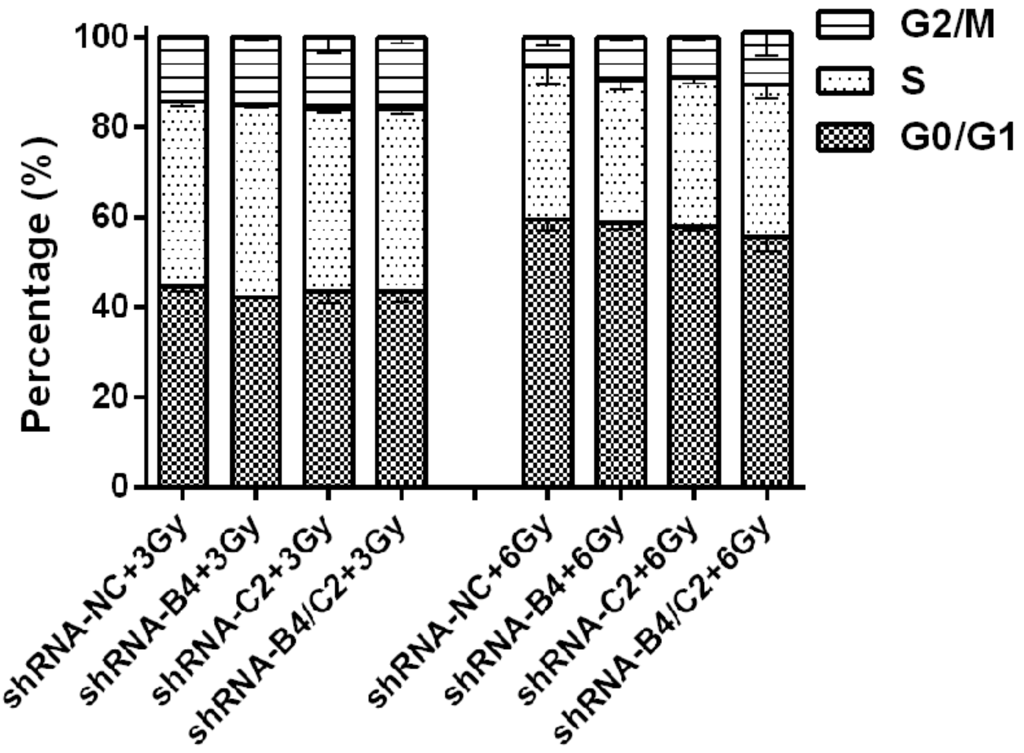

Supplemental Figure 3

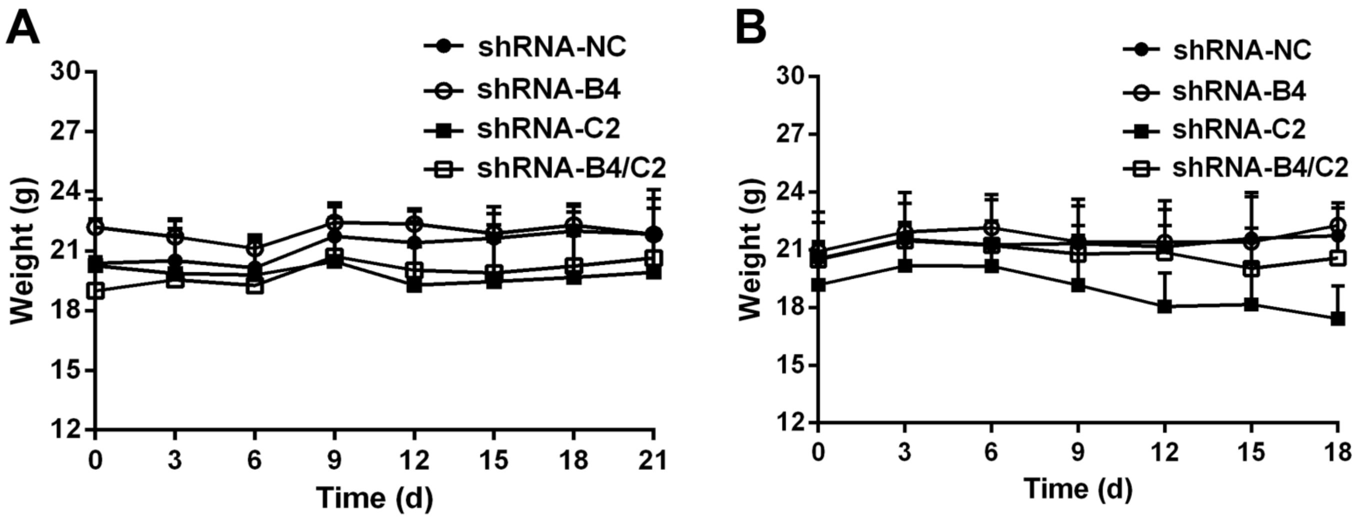

Supplemental Figure 4

Supplemental Table 1 Information of lung cancer patients

Variables	Subgroup	Grade I	Grade II	Grade III	Total
Gender	Male	3	34	5	42 (64.62%)
	Female	5	13	5	23 (35.38%)
Age	Average Age	60	54.5	56.9	58.6
	Age Range	55-69	45-78	32-75	32-78
Histological Features	Adenocarcinoma	0	25	5	30 (46.15%)
	Squamous carcinoma	3	22	5	30 (46.15%)
	Bronchioloalveolar carcinoma	5	0	0	5 (7.70%)

Supplemental Table 2 Targeting sequences for shRNAs

Name	Sequence	Location
shRNA-NC	5’-GTTCTCCGAACGTG-3’	
shRNA-B3	5’-TGGGCACTGCGAATGCCATGACTGA-3’	3-28
shRNA-B4	5’-AATGGCTATAGTGCAGAGTAATGCC-3’	4-29
shRNA-B364	5’-GGTATGCAGATCTTCGTGAAG-3’	364-385
shRNA-B675	5’-GGCCAAGATCCAAGATAAAGA-3’	675-696
shRNA-C187	5’-GCTCATAAGACTCGGCCTTAG-3’	187-208
shRNA-C1138	5’-CTGATCAGCAGAGGTTGATCT-3’	1138--1159
shRNA-C409	5’-GATTTGGGTCGCAGTTCTTGT-3’	409-430
shRNA-C345	5’-GGTGAACGCCGATGATTATAT-3’	345-366

Supplemental Table 3 Primers for RT-PCR analysis

gene	Forword	Reverse
UBB	5’-TGTGGTTTCTGGAAGCCTTT-3’	5’-GCTTGCCTGCAAAGATGAG-3’
UBC	5’-GGGTCGCAGTTCTTGTTTGT-3’	5’-TCCAGCAAAGATCAGCCTCT-3’
GAPDH	5’-GCACCGTCAAGGCTGAGAAC-3’	5’-GGATCTCGCTCCTGGAAGATG-3’

Figure legends

Supplementary Figure 1 The expression level of *UBB* and *UBC* mRNA in 24 paired lung cancer tissue and corrresponding normal tissue samples. RT-PCR was used to analyze the relative *UBB* and *UBC* mRNA level.

Supplementary Figure 2 Ubiquitin mRNA and protein expression in cell lines. (A) RT-PCR and (B) Western blot analysis of the expression of *UBB*, *UBC* gene and ubiquitin protein expression in human lung cancer cell lines (H1299, A549, H460 and H1975) and normal bronchial epithelial cell lines (BEAS-2B and HBE). (C) RT-PCR and (D) Western blot analysis of the expression of *UBB*, *UBC* gene and ubiquitin protein expression in H1299 cells after 0, 3, 6 or 9 Gy X-ray irradiation. Arrows indicate the amplification product of tandem repeat sequence of ubiquitin coding genes.

Supplementary Figure 3 Cell cycle distribution after ubiquitin knock-down combined with irradiation in H1299 cells.

Supplementary Figure 4 (A) Body weights of ubiquitin knockdown. (B) Body weights of ubiquitin knockdown plus irradiation. Body weights were determined every three day. Data are presented as the means ± SEM (* $P < 0.05$).

Supplemental Table 1 Targeting sequences for shRNAs

Supplemental Table 2 Primers for RT-PCR analysis

Supplemental Table 3 Information of non-small cell lung cancer patients

Additional supplemental information (uncropped blots)

Apoptosis

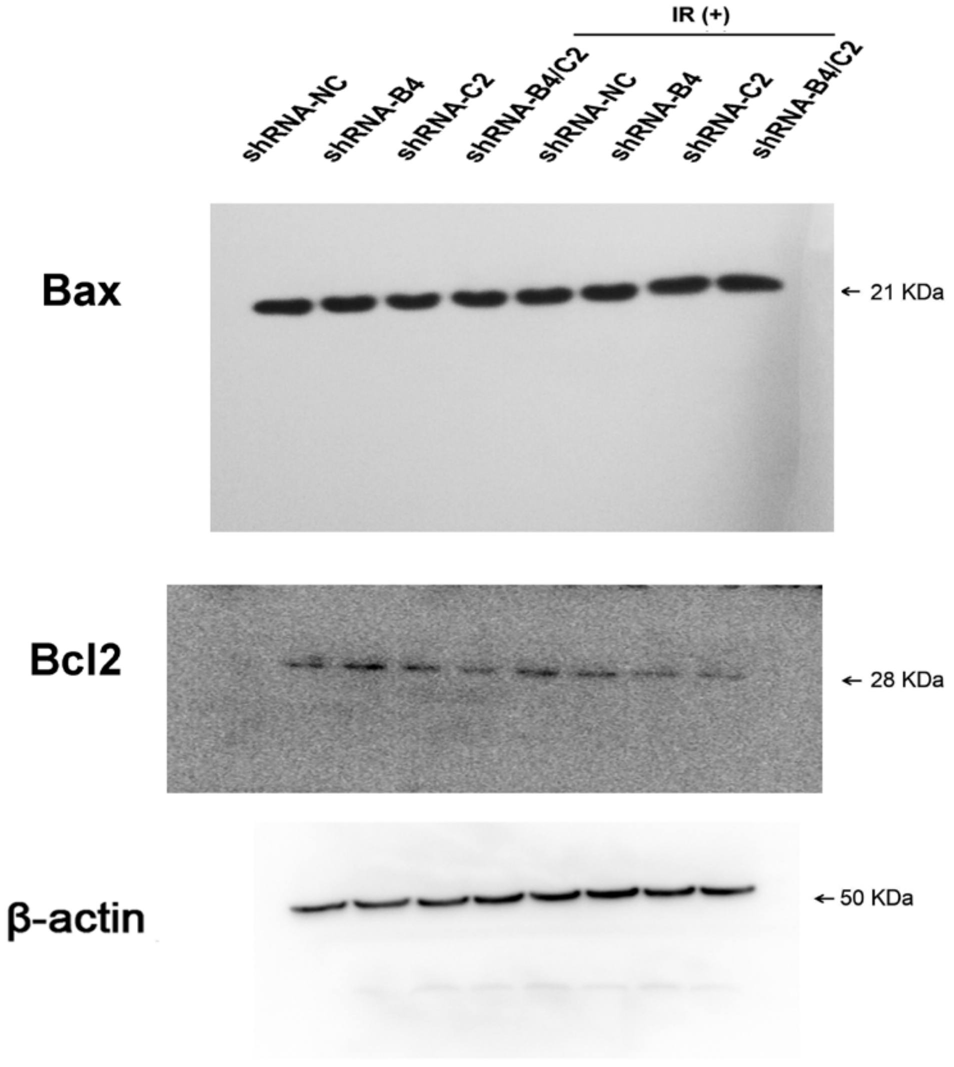

Supplemental Figure 4. Western blot analyze the apoptosis related protein Bcl_2 and Bax.

Cytoplasm protein Western blot

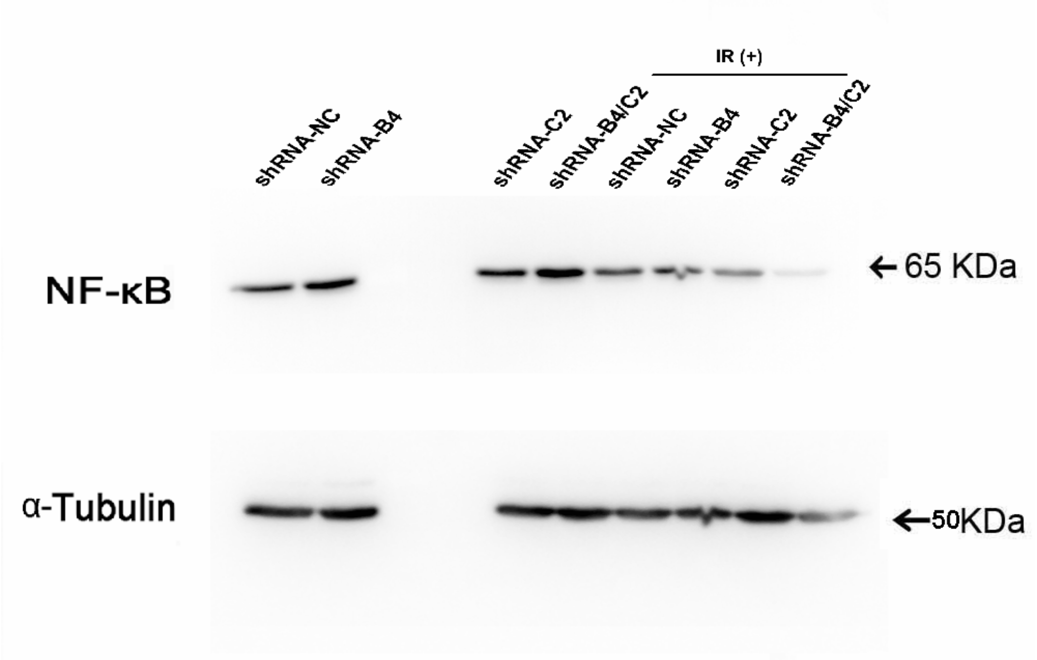

Nucleus Protein Western blot

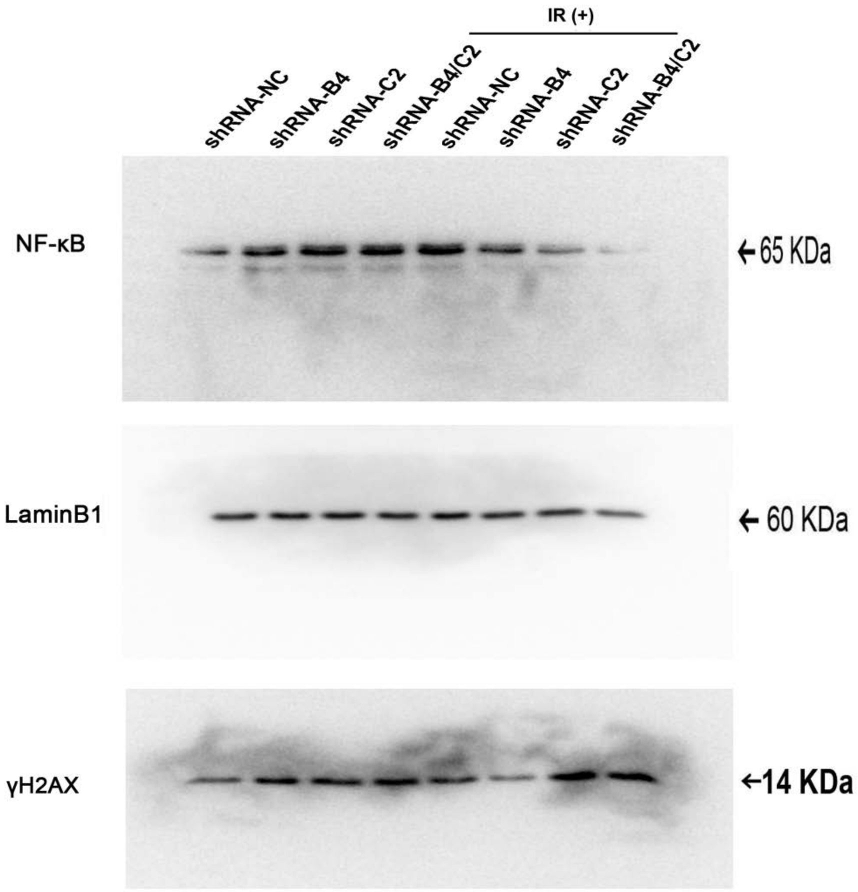

Supplemental Figure 5. Western blot analyze the NF-κB translocate and γH2AX expression

In Vitro

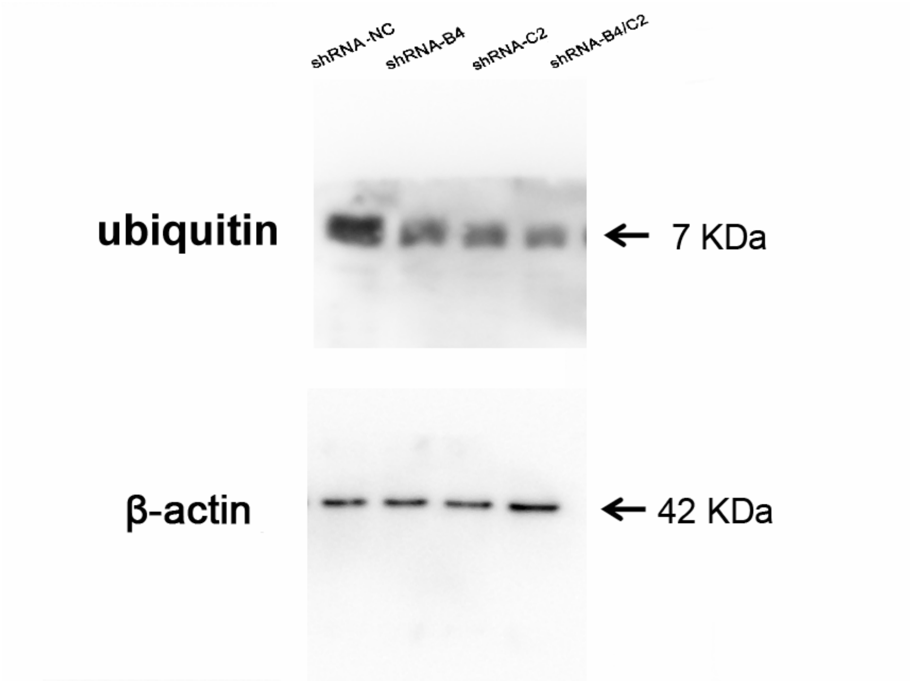

In Vivo

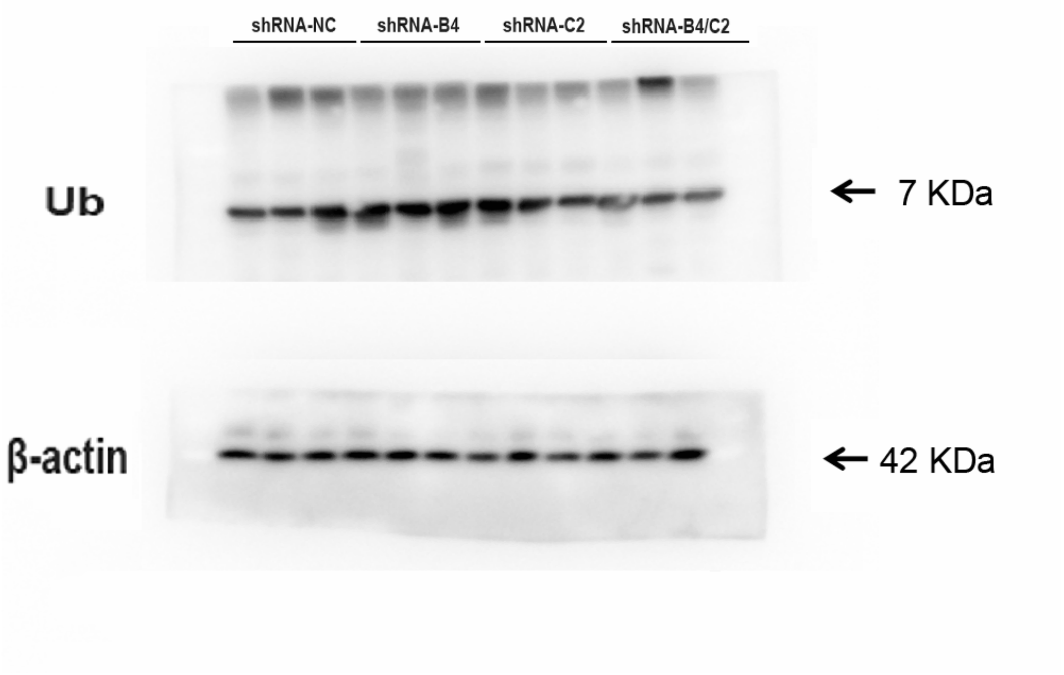

Supplemental Figure 6. Western blot analysis of the shRNA targeting ubiquitin both *in vitro* and *in vivo*

AKT-CyclinD1 pathway

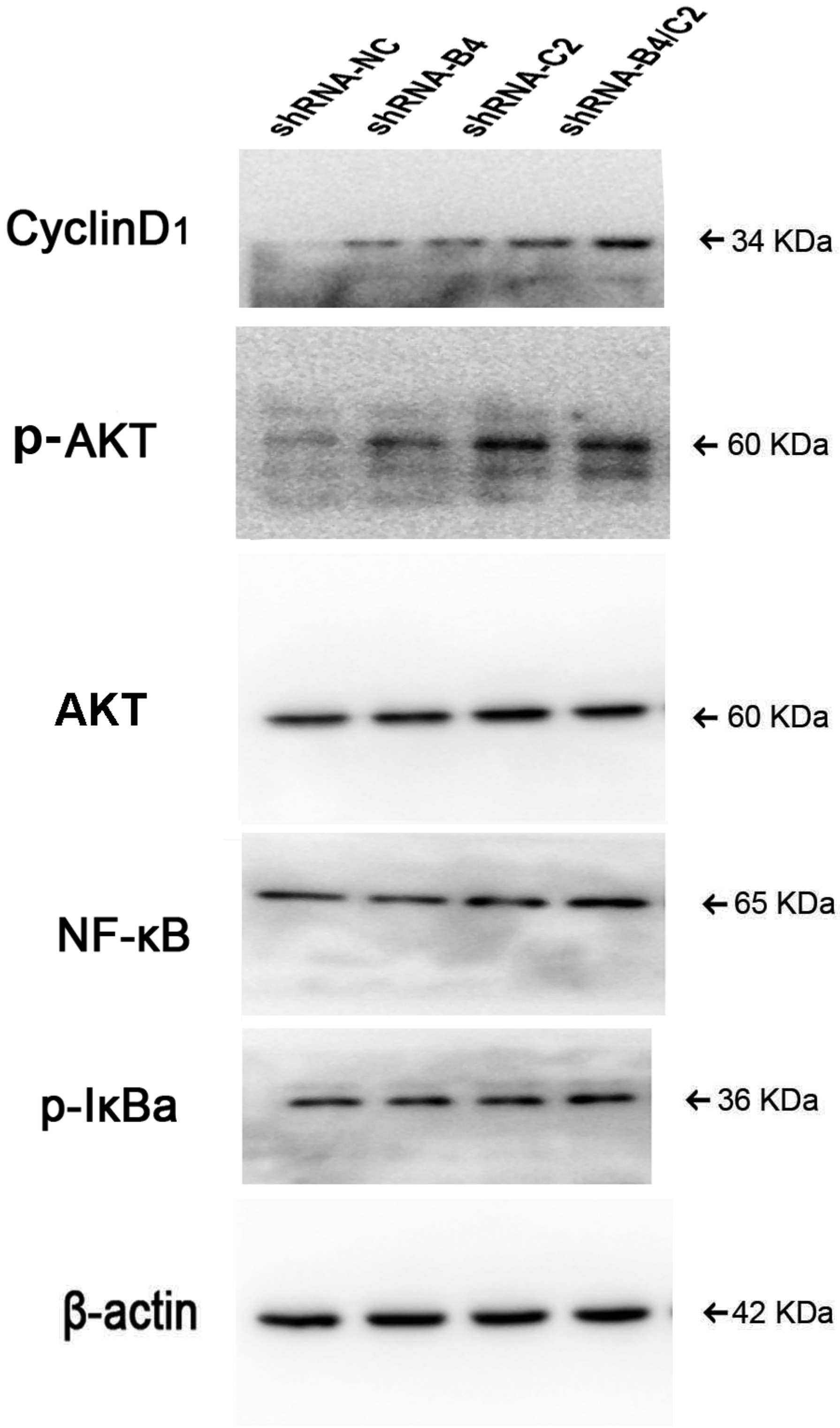

Supplemental Figure 7. ubiquitin inhibition affect the cyclin D1 and phosphor-AKT expression

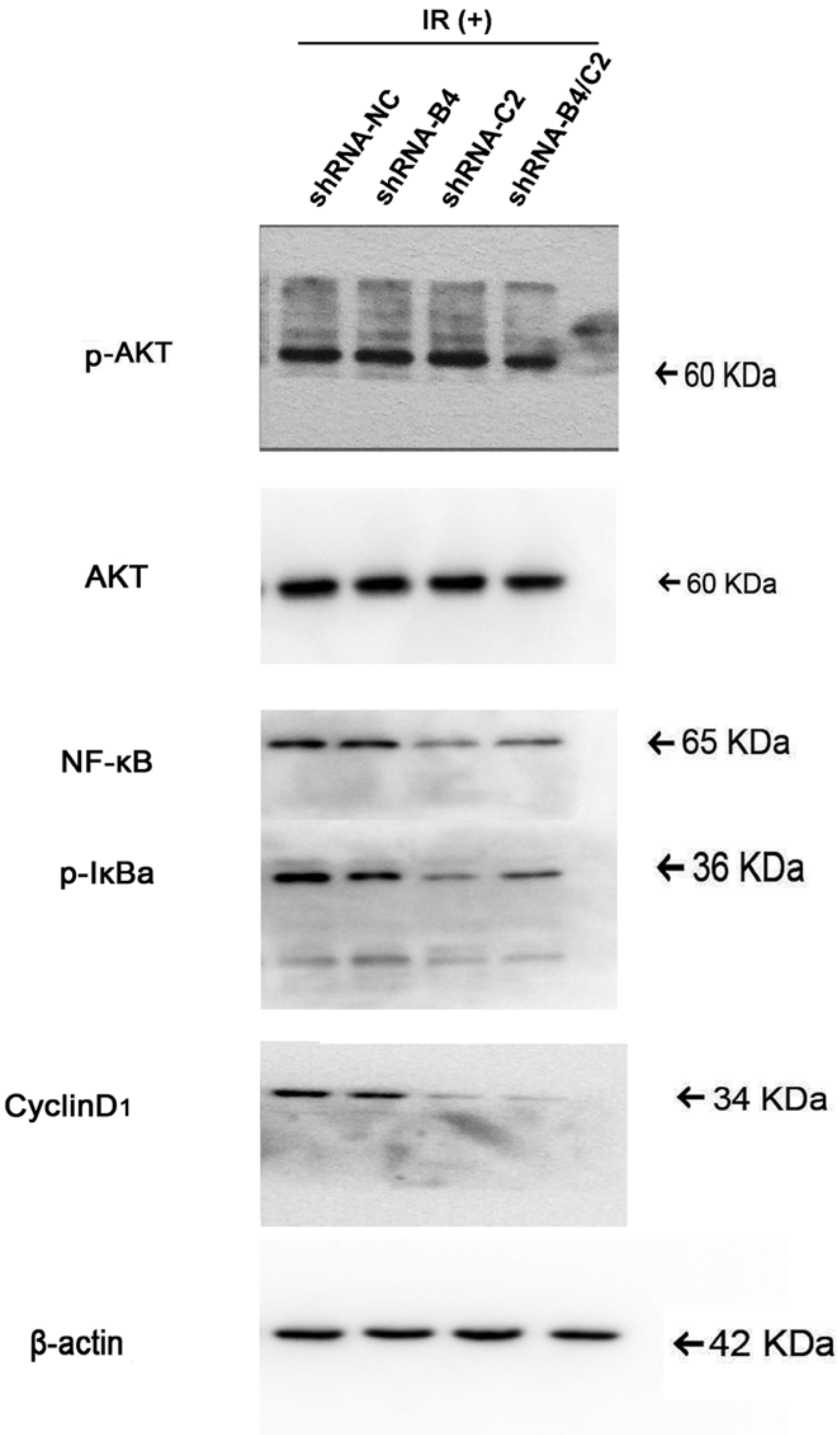

Supplemental Figure 8. Knock-down of ubiquitin affected AKT activation and inhibited NF-κB translocation induced by X-ray irradiation.
